# Linking hnRNP Function to ALS and FTD Pathology

**DOI:** 10.3389/fnins.2018.00326

**Published:** 2018-05-15

**Authors:** Maria D. Purice, J. Paul Taylor

**Affiliations:** ^1^Department of Cell and Molecular Biology, St. Jude Children's Research Hospital, Memphis, TN, United States; ^2^Howard Hughes Medical Institute, Chevy Chase, MD, United States

**Keywords:** amyotrophic lateral sclerosis, frontotemporal dementia, stress granules, hnRNPs, membraneless organelles

## Abstract

Following years of rapid progress identifying the genetic underpinnings of amyotrophic lateral sclerosis (ALS) and related diseases such as frontotemporal dementia (FTD), remarkable consistencies have emerged pointing to perturbed biology of heterogeneous nuclear ribonucleoproteins (hnRNPs) as a central driver of pathobiology. To varying extents these RNA-binding proteins are deposited in pathological inclusions in affected tissues in ALS and FTD. Moreover, mutations in hnRNPs account for a significant number of familial cases of ALS and FTD. Here we review the normal function and potential pathogenic contribution of TDP-43, FUS, hnRNP A1, hnRNP A2B1, MATR3, and TIA1 to disease. We highlight recent evidence linking the low complexity sequence domains (LCDs) of these hnRNPs to the formation of membraneless organelles and discuss how alterations in the dynamics of these organelles could contribute to disease. In particular, we discuss the various roles of disease-associated hnRNPs in stress granule assembly and disassembly, and examine the emerging hypothesis that disease-causing mutations in these proteins lead to accumulation of persistent stress granules.

## Introduction

RNA-binding proteins (RBPs) are a large class of proteins that assemble with RNA to form ribonucleoproteins (RNPs). These proteins largely govern the function and fate of client RNAs, controlling their metabolism at all stages from RNA synthesis (transcription) to degradation (decay). RBPs are among the most abundant proteins in cells, localizing to both the nucleus and cytoplasm, and most are expressed ubiquitously. Heterogeneous nuclear ribonucleoproteins (hnRNPs) are a major subclass of evolutionarily conserved RNPs that are primarily concentrated in the nucleus, although many hnRNPs [e.g., hnRNP A1 and TAR DNA-binding protein 43 (TDP-43)] shuttle between the nucleus and the cytoplasm. The hnRNP family initially consisted of 24 proteins, termed hnRNP A1 through hnRNP U; however, the hnRNP family has grown as well-studied proteins have been later identified as hnRNPs. hnRNPs coat nascent pre-mRNAs to form messenger RNPs (mRNPs), which operate as the functional center of diverse biological processes, including mRNA splicing, polyadenylation, nuclear export, localization, and translation, providing many potential avenues by which hnRNP dysfunction could lead to pathogenesis.

Over the last decade, disturbances in the function of hnRNPs have become closely linked to neurodegenerative diseases, most prominently amyotrophic lateral sclerosis (ALS) and frontotemporal dementia (FTD), two diseases with significant genetic and pathological overlap (Taylor et al., 2016). ALS is a progressive and uniformly fatal neurodegenerative disease characterized by loss of motor neurons in the brain and spinal cord. FTD is a lethal syndrome that results in progressive changes in personality, behavior, and language due to progressive loss of neurons in the frontal and temporal lobes of the brain. Importantly, ALS shows clinical overlap with several other adult-onset degenerative disorders, most frequently FTD, but also inclusion body myopathy (IBM) and Paget's disease of bone (PDB). When manifestations of these conditions (i.e., ALS, FTD, IBM, and/or PDB) are found together, they constitute a clinical syndrome termed multisystem proteinopathy (MSP) (Taylor, 2015).

The first link between hnRNPs and neurodegeneration arose from a 2006 study which recognized that ALS and FTD share a pathological signature defined by prominent deposition of ubiquitin-positive cytoplasmic inclusions that contain TDP-43 (Neumann et al., 2006). Indeed, it is now appreciated that redistribution of TDP-43 from the nucleus to cytoplasmic inclusions is observed in the vast majority (~97%) of sporadic and familial ALS cases, perhaps only absent in those cases involving mutations in *FUS* (fused in sarcoma) or *SOD1* (Cu–Zn superoxide dismutase). TDP-43 pathology is also a prominent feature of virtually all cases of tau-negative FTD (accounting for roughly 50% of FTD cases) (Irwin et al., 2015). In 2007, similar TDP-43 pathology was found also to be a prominent feature of nearly all cases of sporadic and familial IBM (Weihl et al., 2008; Salajegheh et al., 2009). Several years after the appreciation of TDP-43 pathology in ALS, FTD, and IBM, the importance of this feature was underscored by the identification of ALS-causing mutations in the gene encoding this RBP (Gitcho et al., 2008; Kabashi et al., 2008; Kuhnlein et al., 2008; Rutherford et al., 2008; Sreedharan et al., 2008; Van Deerlin et al., 2008; Yokoseki et al., 2008; Pamphlett et al., 2009). Since then, mutations impacting additional RBPs, including FUS, hnRNP A1, hnRNP A2B1, matrin-3 (MATR3), and TIA1, were identified that are causative of ALS, FTD, and/or IBM (Kwiatkowski et al., 2009; Vance et al., 2009; Kim et al., 2013; Liu et al., 2013; Johnson et al., 2014; Mackenzie et al., 2017). Moreover, in these diseases many of these RBPs have been reported to become depleted from the nucleus and deposited in cytoplasmic inclusions.

These findings suggest that perturbed RBP function and associated alteration in RNA metabolism are important drivers of the progression and pathology of sporadic ALS and FTD and could therefore link inherited and spontaneous forms of the disease. However, critical gaps exist in our understanding of the links between neuronal function, dysfunctional RNA metabolism, and other cellular events underlying the pathogenesis of ALS and FTD. Although a growing list of mutations responsible for ALS and FTD impinge on many aspects of RNA metabolism and protein homeostasis (recently reviewed in Taylor et al., 2016; Webster et al., 2017), in this mini-review, we focus on the subset of disease-causing RBPs that are hnRNPs, namely TDP-43, FUS, hnRNP A1, hnRNP A2B1, MATR3, and TIA1.

## Common characteristics of disease-causing hnRNPs

**Figure 1:**
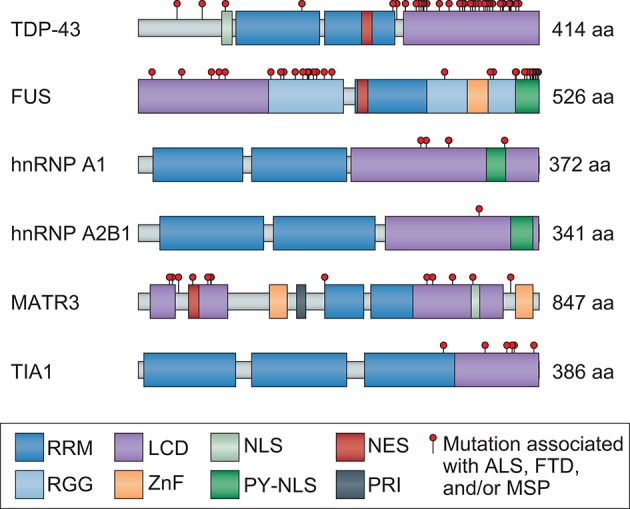
Mutations that cause ALS and FTD cluster in the LCD of hnRNPs and/or disrupt nuclear localization. TDP-43, FUS, hnRNP A1, hnRNP A2B1, MATR3, and TIA1 are ubiquitously expressed hnRNPs that are similar in domain structure and function, each containing at least one RRM and LCD. The LCD region of all hnRNPs is well established with the exception of MATR3, for which PONDR score analysis (http://www.pondr.com/) was used to identify the disordered LCD regions. Mutations identified in patients with ALS, FTD, and/or MSP are depicted as red circles. Mutations in TDP-43, FUS, hnRNP A1, hnRNP A2B1, and TIA1 primarily cluster in the LCD region, whereas mutations in MATR3 are less concentrated in a single region. Several mutations are also found in the NLS of FUS and one in the NLS of TDP-43, causing them to mislocalize to the cytoplasm. MATR3 contains mutations in both the NES and NLS, although further studies are necessary to confirm their effects on MATR3 localization. RRM, RNA recognition motif; LCD, low-complexity domain; NLS, nuclear localization signal; NES, nuclear export signal; RGG, Arg-Gly-Gly box; ZnF, zinc finger; PY-NLS, proline-tyrosine NLS; PRI, polypyrimidine tract binding (PTB)-RRM interaction domain.

Structurally, all hnRNPs contain one or more RNA-binding domains, the most common of which is designated as an RNA recognition motif (RRM) (Figure 1). While all hnRNPs discussed in this review include an RRM, they each contain at least one additional type of RNA-binding domain, defined as a K-homology (KH) domain or a zinc finger motif, both of which facilitate binding to specific RNA sequences, and/or one or more RGG (Arg-Gly-Gly) boxes, which provide strong RNA interaction without a great deal of specificity (Figure 1).

TDP-43, FUS, hnRNP A1, hnRNP A2B1, MATR3, and TIA1 are all ubiquitously expressed and carry out varying functions depending on whether they are localized to the nucleus or the cytoplasm. Such localization is facilitated by both nuclear export sequences and nuclear localization sequences (NLSs), allowing nucleocytoplasmic shuttling (Figure 1). TDP-43 and MATR3 each contain a classic bipartite NLS, and MATR3 also includes a membrane retention signal that can anchor chromosomes to the nuclear matrix (Hibino et al., 1992, 2006; Coelho et al., 2015). In contrast, FUS, hnRNP A1, and hnRNP A2B1 each contain a single proline-tyrosine NLS (PY-NLS), a specialized type of NLS that confers dependence on a single receptor (KapB2 or transportin) (Lee et al., 2006; Dormann et al., 2012; Twyffels et al., 2014; Figure 1). Although detailed analysis of TIA1 has not revealed sequence determinants related to classically understood nuclear localization, the localization of TIA1 is transcription-dependent and its nuclear import and export are mediated by its RRM2/3 domains (Zhang et al., 2005).

**Figure 2:**
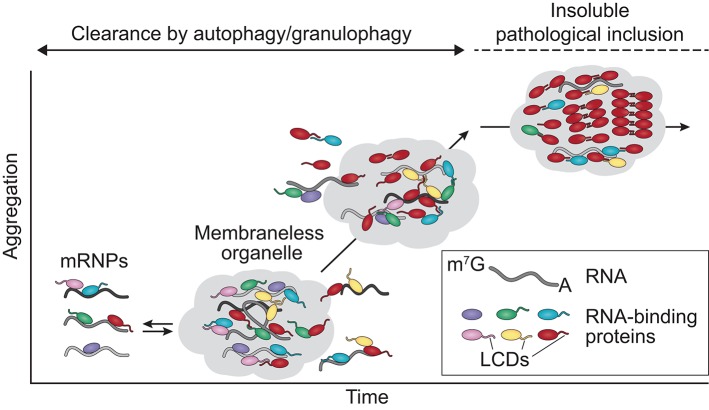
ALS mutations alter the dynamics and function of membraneless organelles. RNA-binding proteins associate with mRNA, forming mRNPs that are transported to the cytosol. The hnRNPs discussed in this review contain LCDs that mediate phase separation, contributing to the assembly/disassembly, dynamics, and liquid properties of membraneless organelles such as stress granules. Stress granules that begin forming aggregates and are no longer capable of being disassembled by liquid-liquid phase separation mechanisms are cleared by autophagy/granulophagy. Disease-causing mutations can trigger the assembly of aberrant or persistent membraneless organelles in which the high concentration and close interactions of the LCDs over time may promote the transition of aggregation-prone proteins (e.g., TDP-43, FUS) to pathogenic amyloid fibrils. mRNP, messenger ribonucleoprotein particle; LCDs, low complexity domains.

Each of the disease-associated hnRNPs discussed in this review also contains at least one low complexity sequence domain (LCD), such as glycine-, proline-, or acid-rich domains: TDP-43 has a C-terminal glycine-rich LCD that mediates interactions with other hnRNPs, including hnRNP A1, hnRNP A2B1, hnRNP A3, and FUS (Buratti et al., 2005; Kitamura et al., 2016); FUS contains an N-terminal SYGQ-rich (serine-, tyrosine-, glycine-, glutamine-rich) LCD that functions in transcriptional activation; hnRNP A1 and hnRNP A2B1 each contain a C-terminal glycine-rich LCD that contains an RGG motif required for stress granule localization; MATR3 has several predicted LCDs; and TIA1 contains a glutamine-rich C-terminal LCD that promotes stress granule assembly (Kedersha et al., 2000; Gilks et al., 2004; Panas et al., 2016). Although historically considered to be protein-protein interaction domains, LCDs contain motifs that can be recognized by other proteins or nucleic acids, resulting in multivalent interactions. This ability to interact with multiple partners is essential for the liquid-liquid phase separation property exhibited by hnRNPs, a biophysical phenomenon that promotes higher order intracellular assemblies, most notably membraneless organelles. These types of organelles are highly relevant to RNA metabolism, as several types of RNP-based membraneless organelles, including nuclear speckles, processing bodies (P bodies), RNA transport granules, and stress granules, have recently emerged as complexes that can regulate RNA metabolism (e.g., RNA splicing, translation, and decay). These structures are of particular interest in the context of highly polarized neurons, some of which have axons 10,000 times longer than their cell bodies, and in which local translation in distinct (and distant) subcellular compartments is necessary for rapid responses to local stimuli. In these neurons, as in many other cell types, nascent mRNA transcripts are sequestered with associated RBPs (e.g., TDP-43, hnRNP A1, and FUS) into RNA granules that inhibit mRNA translation and decay until the granules are delivered to specific sites within the cell (Kanai et al., 2004; Belly et al., 2005; Elvira et al., 2006; Sephton and Yu, 2015). Moreover, TDP-43, FUS, hnRNP A1, hnRNP A2B1, and TIA1 are required for the dynamic assembly, disassembly, and function of stress granules, while MATR3 is important for the formation of P bodies (Rajgor et al., 2016). RNA granules, stress granules, and P bodies are highly dynamic structures that behave like liquid droplets in cells, similar to how oil droplets act when mixed with water. Stress granules can be targeted for degradation by autophagy in a process termed granulophagy. However, as described below, ALS- and FTD-causing mutations in hnRNPs and other RBPs are thought to foster a less dynamic (and typically less soluble) state within stress granules and/or other membraneless organelles, possibly promoting fibrillization of aggregation-prone proteins and likely also adversely affecting RNA metabolism by disturbing their normal functions (Figure 2).

## TDP-43

### Structure and function

A central player in the pathogenesis of ALS/FTD, TDP-43 is a 414-amino acid protein implicated in a wide variety of cellular functions (Figure 1). The amino terminus is composed of a 77-amino acid domain that mediates homodimerization and tetramer formation, an assemblage that appears to be important to its function in pre-mRNA splicing (Jiang et al., 2017). TDP-43 also harbors two tandem RRMs that preferentially bind UG-rich RNA or TG-rich DNA with high affinity (Buratti and Baralle, 2001; Kuo et al., 2014). Biochemical cross-linking studies suggest that TDP-43 may bind to thousands of RNA targets (Polymenidou et al., 2011; Tollervey et al., 2011), indicating that this protein has the potential to impact RNA metabolism on a broad scale. Indeed, early proteomic studies found that TDP-43 strongly interacts with proteins involved in RNA splicing and translation machinery (Freibaum et al., 2010). Since then, evidence has accumulated implicating TDP-43 as influencing RNA metabolism at every stage of the RNA life cycle, including transcription, splicing, mRNA processing, microRNA processing, regulation of coding and long non-coding RNA expression, mRNA transport, mRNA stability, and the formation of cytoplasmic RNA granules (recently summarized in Ratti and Buratti, 2016). As may be expected for a protein that plays a central role in RNA metabolism, levels of TDP-43 are tightly regulated (Ayala et al., 2011; Polymenidou et al., 2011) and, in fact, TDP-43 regulates its own expression by binding to its own 3′ untranslated region (Ayala et al., 2011; Polymenidou et al., 2011; Avendano-Vazquez et al., 2012). Additional RNA-related functions have been revealed by characterization of mouse embryonic stem cells in which TDP-43 has been knocked out, including the notable finding that a lack of TDP-43 leads to retention of cryptic exons that in some cases disrupts translation or promotes nonsense-mediated decay, revealing a role for TDP-43 as a guardian of the transcriptome through repression of cryptic exons (Ling et al., 2015). TDP-43 has also been found to physically associate with the Drosha microprocessor complex, suggesting a role in miRNA biogenesis (Gregory et al., 2004; Ling et al., 2010; Kawahara and Mieda-Sato, 2012). Consistent with this suggestion, a defect in microRNA biogenesis has been reported in neurons derived from patients with TDP-43 mutations (Zhang et al., 2013). Studies of the localization and dynamics of TDP-43 in neurons have reported that cytoplasmic TDP-43 redistributes to axons and dendrites in response to activity and influences neurite outgrowth (Wang et al., 2008; Fallini et al., 2012). Subsequently, TDP-43 was recognized as a constituent of RNA granules that traffic mRNAs to distal compartments for local translation (Wang et al., 2008).

### Role in disease

In both neurons and glia of patients with ALS and/or FTD, TDP-43 is mislocalized from the nucleus to the cytoplasm, where it is heavily post-translationally modified via cleavage, phosphorylation, acetylation, and ubiquitination, and forms granular pathology that evolves to one or a few large inclusions. Because this subcellular redistribution leads to nuclear depletion of TDP-43, the pathogenic mechanism may involve loss of nuclear function, gain of cytoplasmic function, or, most likely, a contribution from both. Currently, more than 40 familial and sporadic mutations in TDP-43 are known to lead to ALS and/or FTD, accounting for an estimated 5% of familial ALS cases and <1% of sporadic cases (Gendron et al., 2013; Taylor et al., 2016). Interestingly, nearly all of the disease-causing mutations are clustered in the LCD region. The LCD of TDP-43 is required for the recruitment of TDP-43 to stress granules and is thought to mediate the assembly of translation-stalling cytoplasmic granules and their liquid properties; furthermore, it influences the solubility and cellular localization of TDP-43 (Ayala et al., 2008; Dewey et al., 2011; Ramaswami et al., 2013). Two disease-causing mutations are located in the RRM and are predicted to generate a more stable protein that is cleaved more efficiently, thereby increasing the amount of cytoplasmic C-terminal fragments of TDP-43 (Austin et al., 2014; Chiang et al., 2016).

Recent *in vitro* studies have demonstrated that within the LCD of TDP-43, a balance between hydrophobic and electrostatic forces results in a conserved α-helical structure that is required for induction of liquid-liquid phase separation; interestingly, ALS mutations within this region significantly disrupt phase separation and promote the conversion to aggregates (Conicella et al., 2016; Li et al., 2018). Consistent with these observations linking the mutation-rich LCD to the biophysical behavior of TDP-43, several recent investigations of disease-causing TDP-43 mutations have yielded important insights into RNP granule dynamics in primary neurons. In these studies, RNP granules consisting of wild-type TDP-43 exhibited distinct biophysical properties depending on their axonal location, suggesting that the properties of TDP-43 granules are dependent on subcellular context and can change over time (Gopal et al., 2017). In contrast, granules formed by ALS-linked mutant TDP-43 are more viscous and show disrupted axonal transport dynamics, suggesting that the increased viscosity observed in TDP-43 mutant granules may lead to a toxic gain of function, possibly enhancing its propensity for aggregation (Alami et al., 2014; Gopal et al., 2017). Disruption in trafficking of RNA granules due to mutant expression of TDP-43 or lack of expression of wild-type TDP-43 also has detrimental consequences for neurons, since local protein synthesis is highly dependent on mRNP granule transport and TDP-43 binds to mRNAs that encode genes related to synaptic development and function (Wang et al., 2008; Godena et al., 2011; Narayanan et al., 2013; Alami et al., 2014; Coyne et al., 2014).

## FUS

### Structure and function

FUS or FUS/TLS (fused in sarcoma/translocated in liposarcoma) is a 526-amino acid protein that shares its classic domain architecture (SYGD domain, three RGGs, one RRM, one zinc finger domain, and a PY-NLS) with EWS and TAF15 proteins, which together with FUS form the FET family of proteins (Figure 1). FUS is primarily localized to the nucleus, although super-resolution imaging of mouse hippocampal neurons has detected FUS in dendritic post-synaptic compartments and within axonal terminals in close proximity to presynaptic proteins and vesicles (Schoen et al., 2015). FUS is involved in diverse cellular processes including cell proliferation (Bertrand et al., 1999), DNA damage repair (Baechtold et al., 1999; Bertrand et al., 1999; reviewed in Sama et al., 2014), and almost every aspect of RNA metabolism, including transcription and stress granule function (Yang et al., 1998; Bentmann et al., 2013). Most recently, FUS has been shown to control back-splicing reactions leading to production of circular RNA (RNA in which the 3′ and 5′ ends have been joined together) in rodent stem cell-derived motor neurons, further adding to the complexity of FUS-dependent functions (Errichelli et al., 2017). Interestingly, FUS has also been found to associate with the Drosha microprocessor (Gregory et al., 2004; Ling et al., 2010; Kawahara and Mieda-Sato, 2012) as well as Dicer complexes (Freibaum et al., 2010; Kawahara and Mieda-Sato, 2012), suggesting a role for FUS (along with TDP-43, which also associates with Drosha) in miRNA biogenesis.

### Role in disease

Three years after the discovery of TDP-43 as the major component of inclusions in the central nervous system of nearly all ALS patients, FUS was identified as the primary component of TDP-43-negative cytoplasmic inclusions in rare patients with ALS, all of whom had causative genetic mutations in *FUS* (Vance et al., 2009; Suzuki et al., 2010; Tateishi et al., 2010). Mutations in *FUS* have been estimated to account for up to 4% and 1% of total familial and sporadic ALS cases respectively (Lagier-Tourenne et al., 2010). FUS pathology is also observed in approximately 10% of FTD cases, although there are no reported cases of FUS-related mutations that lead to FTD (Nolan et al., 2016). The majority of pathogenic FUS mutations are located in the C-terminal PY-NLS or completely delete the NLS sequence, thereby impairing nuclear import of FUS (Dormann et al., 2010), leading to accumulation of FUS in the cytosol and giving rise to an apparent toxic gain of function (Scekic-Zahirovic et al., 2016; Sharma et al., 2016). Mutations in the NLS correlate with both severity of disease and age of onset, such that increased mislocalization to the cytoplasm is associated with earlier onset and more aggressive disease (Dormann et al., 2012; Kuang et al., 2017).

Similar to mutated TDP-43, mislocalized mutant FUS protein expressed in mammalian cell lines or in primary rodent neurons is recruited to stress granules (Bosco et al., 2010; Dormann et al., 2010; Ito et al., 2011; Bentmann et al., 2012; Baron et al., 2013; Lenzi et al., 2015; Kuang et al., 2017). Moreover, biophysical studies have demonstrated that FUS, at physiological concentrations, forms liquid-like droplets that can convert into a solid state, a process of phase conversion that is exacerbated by disease-causing mutations in the LCD (Han et al., 2012; Patel et al., 2015). Taken together, these findings suggest a model in which disease-associated LCD mutations or nuclear import defects cause increased cytoplasmic concentration of FUS and augment its recruitment to stress granules, which over time develop into pathological inclusions, potentially due to increased FUS self-interactions or misfolding (Bentmann et al., 2013; Lin Y. et al., 2015; Molliex et al., 2015; Patel et al., 2015).

## hnRNP A1 and hnRNP A2B1

### Structure and function

Two of the most abundantly expressed proteins in cells, hnRNP A1 is a 372-amino acid protein encoded by *HNRNPA1* and hnRNP A2B1 is a 341-amino acid protein (identical to its isoform hnRNP B1 but lacking 12 amino acids) encoded by *HNRNPA2B1* (Jean-Philippe et al., 2013). hnRNP A1 and hnRNP A2B1 share similar domain architecture and are primarily localized to the nucleus (Nakielny and Dreyfuss, 1999; Jean-Philippe et al., 2013; Kim et al., 2013; Thandapani et al., 2013; Wall and Lewis, 2017; Figure 1). Because the LCDs of both proteins contain steric-zipper motifs, which are two self-complementary beta sheets that can give rise to the spine of an amyloid fibril, these domains are predicted to have high fibrillization propensity (Goldschmidt et al., 2010). Although the two proteins do have some distinct roles in transcriptional control, hnRNP A1 and hnRNP A2B1 have many overlapping functions, including processing of heterogeneous nuclear RNAs into mature mRNAs, splicing, translation, stabilization of mRNA, transcriptional elongation, and regulation of DNA metabolism associated with telomeres (Beriault et al., 2004; Zhao et al., 2009; Flynn et al., 2011; Guo et al., 2013; Jean-Philippe et al., 2013; Lemieux et al., 2015). Notably, hnRNP A1 and hnRNP A2B1 are components of RNA transport granules in neurons (Elvira et al., 2006). In addition, hnRNP A1 and hnRNP A2B1 translocate to the cytoplasm in response to stress and are recruited to stress granules (Guil et al., 2006; McDonald et al., 2011).

### Role in disease

Mutations in the aggregation-prone LCDs of hnRNP A1 and hnRNP A2B1 account for <1% of familial and sporadic forms of ALS and are more frequently associated with the broader spectrum disorder MSP (Kim et al., 2013; Taylor, 2015). Interestingly, muscle biopsies of MSP patients have revealed concurrent cytoplasmic mislocalization/nuclear clearing and partial colocalization of TDP-43 and hnRNP A1 or TDP-43 and hnRNP A2B1 (Kim et al., 2013). Disease-causing mutations are predicted to strengthen the steric zippers of hnRNP A1 and hnRNP A2B1, altering the dynamics of stress granule assembly and accelerating nucleation and fibrillization *in vitro* (Kim et al., 2013). Consistent with these predictions, *in vitro* synthetic studies have shown that both wild-type hnRNP A1 and hnRNP A2B1 form fibrils and that disease mutations greatly enhance this fibrillization. Furthermore, mutated hnRNP A1 and hnRNP A2B1 form fibrils that are self-seeding and can recruit wild-type protein, suggesting that these proteins have prion-like properties (Kim et al., 2013). In a more detailed study of hnRNP A1, the LCD was demonstrated to drive the phase separation that mediates the assembly of stress granules and their liquid properties. In these experiments, RNA binding contributed to phase separation and enhanced fibrillization in protein-rich droplets (Molliex et al., 2015). These findings suggest that hnRNP A1 acts in a concentration-dependent manner and interacts with RNA to mediate phase transition and drive the formation of membraneless organelles. Furthermore, since disease-causing mutations in hnRNP A1 promote fibrillization *in vitro*, mutations that alter the dynamics of membraneless organelles could result in accelerated fibrillization and the formation of aggregates that can accumulate within the cell (Molliex et al., 2015).

## MATR3

### Structure and function

MATR3 is an 847-amino acid protein encoded by the *MATR3* gene and consists of two RRMs flanked by two zinc finger domains and a conserved polypyrimidine tract binding-RRM interaction (PTB-RRM; PRI) domain (Figure 1). MATR3 is predicted to have extensive LCDs, and PONDR (Predictor Of Naturally Disordered Regions) score analysis identifies three disordered LCD regions of 50 or more amino acids in length (Romero and Dunker, 1997; Garner et al., 1998, 1999; Coelho et al., 2015; Figure 1). Although MATR3 is primarily localized to the inner nuclear matrix and nucleoplasm, it has also been detected in the cytoplasm at low levels (Hibino et al., 2006). MATR3 has several functions, including roles in transcription (Skowronska-Krawczyk and Rosenfeld, 2015) and early stages of the DNA damage response (Salton et al., 2010). MATR3 also functions in RNA metabolism, including mRNA stabilization (Salton et al., 2011), the coupling of transcription to splicing via interaction with RNA polymerase II (Das et al., 2007), nuclear retention of hyperedited RNAs to prevent their translation (Zhang and Carmichael, 2001), and splicing (Coelho et al., 2015). In addition, MATR3 interacts via RNA with other hnRNPs, including TDP-43 and hnRNP K, potentially to regulate several RNA processes including transcription and splicing (Salton et al., 2011; Johnson et al., 2014).

### Role in disease

Mutations in *MATR3* account for approximately 1% of total cases of ALS (Johnson et al., 2014; Lin K. P. et al., 2015; Origone et al., 2015; Leblond et al., 2016; Xu et al., 2016; Marangi et al., 2017) and are also causative of autosomal dominant late-onset distal myopathy (Muller et al., 2014). In both sporadic and familial cases, MATR3 binds directly to TDP-43, and at least some disease-causing mutations alter this binding in a mutation-selective and RNA-dependent manner (Johnson et al., 2014). In healthy human tissue, MATR3 is found in a granular pattern in the nuclei of motor neurons and surrounding glial cells. In patients with disease-causing ALS mutations, MATR3 is largely localized to the nucleus, with occasional immunostaining in the cytoplasm (Johnson et al., 2014). These findings parallel a cellular model that showed that ALS/myopathy-associated MATR3 mutations do not produce profound changes in the localization of MATR3 (Gallego-Iradi et al., 2015). However, recently evidence has demonstrated that MATR3 is a component of neuronal cytoplasmic inclusions in motor neurons in cases of sporadic ALS (Tada et al., 2017). Surprisingly, unlike the other hnRNPs discussed in this review, stressed cells overexpressing wild-type or mutant MATR3 do not recruit MATR3 to stress granules or change the nuclear localization of MATR3, and do not induce the formation of inclusion-like structures in either the cytoplasm or nucleus (Gallego-Iradi et al., 2015). Although the mechanisms by which MATR3 might lead to ALS pathology remain to be elucidated, it appears that alterations in expression levels of MATR3 in the muscle and spinal cord might be critical for neuromuscular function and that dysregulation of its expression may underlie some aspects of neuromuscular dysfunction (Moloney et al., 2016; Rayaprolu et al., 2016). In addition, recent data suggest that expression of disease-causing MATR3 mutations leads to defects in nuclear export of global mRNA and more specifically mRNA encoding TDP-43 and FUS, potentially contributing to pathogenesis (Boehringer et al., 2017).

## TIA1

### Structure and function

TIA1 is a 386-amino acid protein that is structurally very similar to TDP-43 (Figure 1). Notably, each of its three RRMs has a distinct RNA-binding profile (Dember et al., 1996; Forch et al., 2002; Kim et al., 2007; Bauer et al., 2012; Wang et al., 2014). RRM1 binds AU-rich pre-mRNA sequences and interacts with the LCD to recruit and stabilize the U1 small nuclear ribonucleoprotein-associated protein U1C, a splicing regulator (Forch et al., 2002). RRM2 is necessary and sufficient for TIA1 to bind RNA and exhibits the highest binding affinity to U-rich pre-mRNA sequences, and RNA binding is further enhanced when RRM3 is present (Bauer et al., 2012). TIA1 is largely localized to the nucleus and has been associated with several functions related to RNA metabolism, including alternative splicing, translational repression, and mRNA silencing (Lopez de Silanes et al., 2005; Carrascoso et al., 2014; Waris et al., 2014). In response to cellular stress, TIA1 translocates to the cytoplasm, suppresses mRNA translation by binding to specific mRNA transcripts marked by AU-rich elements, and nucleates stress granule formation through self-association of its LCD (Gilks et al., 2004; Lopez de Silanes et al., 2005; Wang et al., 2014; Waris et al., 2014).

### Role in disease

One of the most recent genes associated with ALS, *TIA1* harbors several ALS- and ALS/FTD-associated mutations in its LCD (Mackenzie et al., 2017). Notably, the LCD of TIA1 is also the site of a mutation that causes Welander distal myopathy, a myopathy characterized by TDP-43-positive inclusions (Klar et al., 2013). These disease mutations alter the biophysical properties of TIA1 by significantly increasing its propensity toward phase separation, delaying stress granule disassembly following removal of stressful stimuli, and promoting the accumulation of non-dynamic stress granules that sequester TDP-43 (Mackenzie et al., 2017). Furthermore, TDP-43 recruited to these stress granules becomes immobile and insoluble. Interestingly, although TIA1 is similar in structure and function to TDP-43, examination of patient pathology has revealed inclusions that are immunoreactive for TDP-43 but not TIA1 (Mackenzie et al., 2017). These findings reinforce the importance of disturbed RNA metabolism in neurodegenerative disorders and place dysfunctional membraneless organelle dynamics at the center of disease progression and pathogenesis.

## RBP-mediated pathogenic mechanisms in ALS and FTD

Disease-causing mutations in TDP-43, FUS, hnRNP A1, hnRNP A2B1, MATR3, and TIA1 point to disturbed biology of RBPs, especially hnRNPs, as playing a central pathogenic role in ALS and FTD. This view is further supported by evidence that alleles of the RBP ataxin-2 (ATXN2) with intermediate repeat expansion increase the risk of ALS-FTD, as well as evidence that variants in RBPs not discussed in this review (e.g., ANG, EWS, TAF15) may contribute to the burden of ALS-FTD. All of these RBPs have related domain architecture that includes multiple RNA-binding domains and sizeable LCDs that are predicted to be intrinsically disordered (Figure 1). Notably, for most of these proteins there is a tendency for disease-causing mutations to impact the composition of the LCD, which is a domain that strongly promotes liquid-liquid phase separation to drive the assembly of membraneless organelles such as stress granules (Figure 1). It is important to point out, however, that these RBPs engage in a variety of additional assemblies that arise through phase separation, for example the nucleolus (Berry et al., 2015), P granules (Elbaum-Garfinkle et al., 2015), nuclear speckles (Hennig et al., 2015), and others, and that disease-causing mutations that disturb phase transitions are likely to have a broad impact on cellular physiology that stretches beyond alterations to stress granule dynamics.

Recent findings related to mutations in chromosome 9 open reading frame 72 (*C9ORF72*) have reinforced the importance of phase separation in disease (DeJesus-Hernandez et al., 2011; Renton et al., 2011; Majounie et al., 2012). The leading cause of sporadic and familial ALS, mutations in C9ORF72 lead to disease when an intronic GGGGCC hexanucleotide repeat is massively expanded. In patients with these mutations, the expanded GGGGCC repeats are translated into five different species of dipeptide repeat proteins (DPRs). Two species of these repeats (polyGR and polyPR) feature alternating arginine residues, which are charged, highly polar, and carry a dipole moment. Arginine residues frequently engage in charge-charge interactions with basic residues or Pi-cation interactions with aromatic residues, two interaction types that are thought to contribute significantly to intermolecular adhesion between LCDs to permit phase separation. Notably, the arginine-containing DPRs (polyGR and polyPR) insinuate into and disturb the properties of membraneless organelles, including stress granules, Cajal bodies, nuclear speckles, and the nucleolus—all organelles that are active liquids and are mediated by phase separation (Lee et al., 2016; Boeynaems et al., 2017). Not surprisingly, the arginine-containing DPRs are consistently reported to be the most toxic in living cells (Kwon et al., 2014; Mizielinska et al., 2014; Wen et al., 2014; Freibaum et al., 2015; Lee et al., 2016). Further evidence suggesting a role for arginine-containing DPRs comes from a recent report revealing correlation between the regions of polyGR deposition and neurodegeneration in the brains of patients with C9ORF72-related ALS-FTD (Saberi et al., 2017).

Additional evidence that disturbance in the dynamics of membraneless organelles, especially stress granules, are a unifying mechanism between the many disease-causing RBPs and ALS and/or FTD pathogenesis can be found in the study of disease genes not traditionally linked to RBP biology. For example, mutations in valosin-containing protein (VCP) cause both ALS and FTD. VCP has emerged as an essential factor in the autophagic degradation of stress granules and, indeed, ALS-FTD-causing mutations lead to accumulation of poorly dynamic, TDP-43-positive stress granules (Buchan et al., 2013). Moreover, an enhancer/suppressor screen in *Drosophila* VCP mutants identified three RBPs (TDP-43, hnRNP A1, hnRNP A2B1) that suppressed the mutant VCP-associated phenotype when knocked down (Ritson et al., 2010). As discussed above, all three of these proteins lead to ALS when mutated, all three contain LCDs, and all three are associated with stress granules. In a separate screen of DPR toxicity, proteomic analysis of DPRs identified several DPR interactors, including TDP-43 and ATXN2, another ALS-related RBP that contains an LCD and is recruited to stress granules. TDP-43 and ATXN2 were found to interact genetically with DPRs, as knockdown of these genes suppressed the DPR-associated viability phenotype in flies (Lee et al., 2016). The observations that knockdown of disease-causing LCD-containing RBPs (i.e., TDP-43, ATXN2, hnRNP A1, hnRNP A2B1) suppresses the toxicity caused by other disease-associated proteins (i.e., VCP and DPRs) demonstrates that although disease-causing RBPs have separate functions, they are constituents of stress granules and normally maintain the material properties (i.e., assembly/disassembly rates, mobility, and viscosity) of stress granules, further strengthening the hypothesis that stress granules are dysregulated in ALS and FTD.

Taken together, an emerging refrain of these studies suggests that the consequence of disease-causing mutations in RBPs is a perturbation in the material properties of RNA granules due to increased adhesive forces between constituent proteins, thus increasing the viscosity of these liquid assemblies. This disturbance is expected to have two potentially adverse consequences. First, increased viscosity results in impaired dynamics of RNA granules (i.e., the ability to exchange components with the surrounding cytoplasm or to unpack their RNA cargo at the appropriate time and place) and impaired normal function. Consistent with this hypothesis, mutations in hnRNP A1, hnRNP A2B1, and TIA1 increase the adhesive forces that drive higher order assembly (Kim et al., 2013; Mackenzie et al., 2017), alter the material properties and dynamics of RNA granules composed of these proteins (Mackenzie et al., 2017), and produce poorly dynamic RNA granules (Kim et al., 2013; Mackenzie et al., 2017). Second, the accumulation of poorly dynamic RNA granules is likely to promote longer length-scale (and thus more stable) assemblies of fibrillization-prone proteins such as TDP-43 and FUS. According to this view, persistent, poorly dynamic RNA granules may evolve over time into pathological inclusions that are appreciated in end-stage disease, thus accounting for the frequent association of RNA granule components with ALS-FTD pathology (Figure 2)—a hypothesis that remains to be tested.

## Future questions

Although immense progress has been made toward defining the genetic and biological basis of ALS and FTD, it remains challenging to define the molecular mechanisms that link specific disease-causing mutations to stress granule dysfunction and the accumulation of pathological inclusions. Given the converging evidence described here, one of the most compelling hypotheses is founded on the notion that impairment of the material properties of stress granules leads to impairment of the functions supported by these organelles (Figure 2). At the same time, the residence time of aggregation-prone proteins in the highly concentrated environment is prolonged, increasing the opportunity for nucleating the assembly of stable, amyloid structures and also promoting their growth and maturation (Figure 2). Perhaps the most stable of these is TDP-43, which may be prone to form stable assemblies and become toxic within poorly dynamic stress granules. Going forward, there are four pressing enigmas to resolve that relate to how assemblies built from hnRNPs (such as stress granules) contribute to disease. First, we must define precisely how disease mutations impair the material properties of membraneless organelles. Second, we must identify which function(s) of membraneless organelles are impaired in a manner that contributes to ALS and FTD pathology. Third, in a related question, we need to determine which membraneless organelles are important in this process, including delineating whether stress granules are indeed the key target. Finally, we must define whether impaired membraneless organelles (e.g., stress granules) are the source of TDP-43 pathology, and if so, whether this contributes to driving the pathogenic process. Answering these questions will advance our understanding of the key molecular processes that drive ALS and FTD with the long-term goal of identifying potential targets or processes for therapeutic intervention.

## Author contributions

All authors listed have made a substantial, direct and intellectual contribution to the work, and approved it for publication.

### Conflict of interest statement

The authors declare that the research was conducted in the absence of any commercial or financial relationships that could be construed as a potential conflict of interest.
